# Editorial: Cofactor regeneration technologies for microbial cell factory

**DOI:** 10.3389/fmicb.2023.1196002

**Published:** 2023-04-28

**Authors:** Zhen Fang, Narenkumar Jayaraman, Yongkun Lv, Zhengshan Luo

**Affiliations:** ^1^School of Emergency Management, School of Environment and Safety Engineering, Biofuels Institute, Jiangsu University, Zhenjiang, China; ^2^Department of Environmental and Water Resources Engineering, School of Civil Engineering (SCE), Vellore Institute of Technology, Vellore, Tamil Nadu, India; ^3^School of Chemical Engineering, Zhengzhou University, Zhengzhou, China; ^4^Key Laboratory of Carbohydrate Chemistry and Biotechnology, Ministry of Education, School of Biotechnology, Jiangnan University, Wuxi, China

**Keywords:** microbial cell factory, cofactor regeneration, microbial metabolism, dehydrogenase and hydrogenase, NADH

This Research Topic is part of the Insights in Frontiers in Microbiology series launched in 2022. Cofactors such as NAD(P)^+^, NAD(P)H, FADH_2_, and ATP are frequently seen to couple with microorganism metabolic pathways and intracellular redox reactions. These cofactors are essential for adjusting cell growth and metabolism, as well as non-natural chemical biosynthesis. The microbial cell factory (MCF) usually depends on precise regulation of cofactor regeneration to produce valuable chemicals at industrial fermentation levels. For example, MCFs can utilize one carbon compound (CO_2_, methanol, formate, or methane) as a sole substrate to produce complicated multi-carbon compounds, which highlights the attractive and impressive potential of carbon neutralization. Recently, researchers have been paying more attention to cofactor regeneration since it needs extra electron donors or energy sources to disturb the target pathway. This raises the question of how to efficiently adjust cofactor regeneration in MCFs. Before giving a satisfactory answer, investigations of inherent mechanisms regarding various cofactors connected to microbial metabolism should be conducted. Therefore, this Research Topic aimed to inspire, inform, and provide direction and guidance to researchers in the field of microbial cofactor regeneration.

We are pleased to note that our Research Topic has attracted one review and three research articles.

Intracellular cofactor consumption shows a significant relationship to microbial physiological activity and survival under extreme environments. Yang et al. used *Acetobacter pasteurianus* CGMCC 1.41, a widely used vinegar-brewing strain, as a research model to reveal the cell acid tolerance mechanism, which is related to the down-regulation of NADH, FADH_2_, and pyrroloquinoline quinine (PQQ)-dependent dehydrogenases. RNA-Seq transcriptomic analysis revealed that the above reducing cofactors could reduce electron transfer from the cytoplasm, which contributed to the conversion of ethanol to acetate and increased cell acid resistance. It also indicated that ethanol respiration, as the main energy source, is competitive to the NADH dehydrogenase and succinate dehydrogenase–based respiratory chain. By understanding the acid resistance mechanism it is possible to harness cofactor engineering to guide bacteria to overcome harsh and complicated industrial fermentation processes.

NADH-involved electron bifurcation based on an Rnf complex was previously proposed in anaerobic bacteria such as *Syntrophus aciditrophicus*. Losey et al. provided an alternative possibility that hydrogen production via [FeFe]-hydrogenase stems from NADH without electron bifurcation or the involvement of ferredoxin. In this way, *S. aciditrophicus* will save more energy without using the Rnf complex, which can oxidize NADH molecules to produce reduced ferredoxin. The NADH-involved mechanism may explain the obligatory requirement that many syntrophic metabolizers have for a hydrogen-using partner microorganism when grown on fatty, aromatic, and alicyclic acids.

Wu et al. introduced a highly efficient cellulolytic bacterial strain, *Cellulomonas iranensis* ZJW-6, which was isolated from paddy soil in central China. ZJW-6 has an excellent ability to degrade cellulose, hemicellulose, and lignin, with cellulose and hemicellulose loss rates reaching almost 50% in 4 days and lignin loss rates reaching nearly 30% under aerobic and anaerobic conditions. Experiments to determine enzymatic properties showed that bacterial cellulolytic activities are related to cofactor-dependent enzymes such as lignin peroxidase, manganese peroxidase, and laccase. Owing to cofactor-coupled cellulolytic advantages, this strain showed enormous application potential to improve soil fertility (available phosphorus, available nitrogen, organic matter) and rice growth.

The fourth article is a review that summarizes the state of the art of cofactor regulation in microbial metabolism. Cofactors are key molecules that maintain the cell redox balance and drive cell synthesis and catabolic reactions. Cofactors are involved in almost all enzyme activities occurring in living cells. In recent years, the use of appropriate techniques to manage intracellular cofactor concentration and formation, to achieve higher quality target products, has been a hot Research Topic. Sun et al. comprehensively listed the physiological functions of common cofactors (such as acetyl coenzyme A, NAD(P)H/NAD(P)^+^, and ATP/ADP), detailed intracellular cofactor regeneration pathways, cofactor regulation, and cofactor application progress. The review highlights that cofactor engineering may maximize and rapidly reconstruct the metabolic flux to target metabolites in MCFs.

This Editorial summarizes the articles published in this Research Topic. We hope that this Research Topic of articles will contribute to the advancement of research in microbial cofactor regeneration, especially that related to MCFs. Moreover, cofactor regeneration is necessary to adjust precise biochemical reactions that may be extended to P450 monooxygenase–related pharmaceutical biosynthesis, biofuel production, carbon bio-fixation, and so on. Emerging research related to photo/electrochemical biocatalytic technologies also needs to combine cofactor regeneration *in vivo* or *in vitro*. We hope that the sustainable bioprocess of cofactor regeneration will be a green biological manufacturing technology that can substitute the traditional chemical industry and produce more sustainable chemicals as well as realize carbon neutrality.

Finally, we want to thank all authors who contributed their original work to our Research Topic and the reviewers for their valuable comments.

## Author contributions

ZF drafted this manuscript. YL, ZL, and NJ revised it. All authors contributed to the article and approved the submitted version.

